# Machine learning to predict postdialysis fatigue in patients undergoing hemodialysis

**DOI:** 10.1080/0886022X.2025.2529452

**Published:** 2025-07-27

**Authors:** Yuhan Zhang, Jue Guo, Na Yang, Xiangyun Li, Yuxiang Liu, Meiqin Yan, Peng Shen

**Affiliations:** a College of Nursing, Shanxi Medical University, Shanxi, China; b The Blood purification Department of Shanxi Provincial People’s Hospital, Shanxi, China; c Nursing department, Taiyuan Central Hospital, Shanxi, China; d Department of Nephrology, Shanxi Provincial People’s Hospital, Taiyuan, Shanxi, China; e Department of Nephrology, The Fifth Clinical Medical College of Shanxi Medical University, Taiyuan, Shanxi, China; f Shanxi Provincial Key Laboratory of Kidney Disease, Taiyuan, Shanxi, China; g Science and Education Department of Shanxi Children’s Hospital, Shanxi Medical University, Shanxi, China

**Keywords:** Postdialysis fatigue, hemodialysis, machine learning, prediction model, risk factor

## Abstract

**Background:**

Machine learning (ML) has been widely used to predict complications and prognosis in patients undergoing hemodialysis (HD). However, accurate and efficient models for predicting postdialysis fatigue (PDF) in this population are still needed because PDF is surprisingly prevalent.

**Aims:**

This study aimed to explore the potential of ML models for predicting PDF in patients undergoing HD.

**Data sources:**

A total of 1,281 Chinese patients undergoing HD from six tertiary hospitals (65.26% male, mean age = 54.48 years).

**Design:**

Cross-sectional study.

**Methods:**

Seven ML models were compared: Logistic regression (LR), Decision tree (DT), Random forests (RF), LightGBM (LGBM), CatBoost, XGBoost (XGB), and Gradient boosting tree (GBT), to predict the PDF and identify variables with predictive value based on the best-performing model among Chinese patients undergoing HD. The study findings were reported in accordance with the TRIPOD+AI guidelines.

**Results:**

The RF model achieved the relatively optimal and stable performance, with an area under the curve of 0.855, accuracy of 0.773, F1 score of 0.769, and Brier score of 0.155 in test set. Resilience, appetite, potassium levels, sleep quality, constipation, history of fistula surgery, diastolic blood bressure, and the category of “combined other diseases” were the strongest predictors of PDF.

**Conclusion:**

ML models can serve as convenient screening and assessment tools for PDF risk in Chinese patients undergoing HD. In combination with the SHapley Additive exPlanations (SHAP) approach, the proposed framework provides a more intuitive and comprehensive interpretation of the predictive model, thereby allowing clinicians to better understand the decision-making process of the model and impact of the factors associated with PDF.

## WHAT DOES THIS PAPER CONTRIBUTE TO THE WIDER GLOBAL CLINICAL COMMUNITY?

- Our study succeeded in differentiating PDF from the broader cumulative experience of fatigue in terms of outcome indicators, including chronic and interdialytic fatigue.
- Seven machine learning algorithms were used to construct the prediction model, and RF performed the best.
- This study identified resilience, appetite, potassium levels, sleep quality, constipation, HFS, DBP, and COD as having the highest values for predicting PDF. With the advent of artificial intelligence (AI), dialysis machines can be embedded in the module of the ML prediction model to achieve early intervention in the early warning of PDF.

## Graphical Abstract

**Figure:**
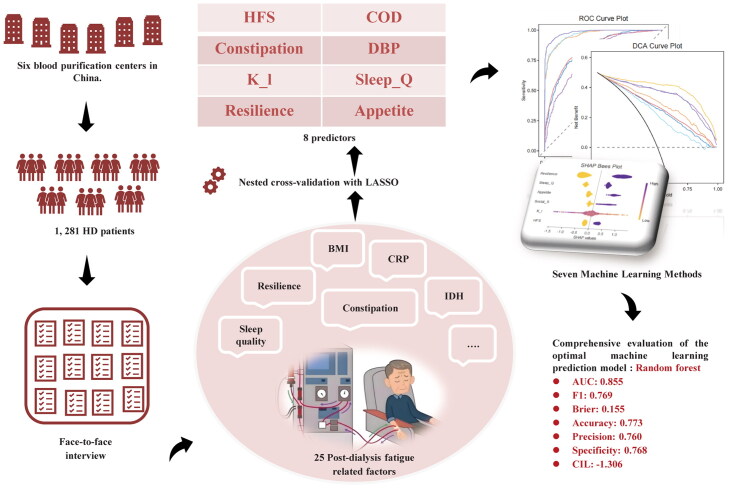

## 1. Introduction

At least one in ten people worldwide has kidney disease [1]. According to the Global Burden of Disease (GBD), more than 3.1 million deaths in 2019 were attributed to kidney dysfunction, making it the seventh leading global risk factor for mortality [2]. In the United States, Medicare fee-for-service spending for beneficiaries with chronic kidney disease (CKD) reached $86.1 billion in 2021, accounting for 22.6% of total healthcare expenditures [3]. These high global mortality rates and significant economic burdens highlight disparities in the prevention, early detection, diagnosis, and treatment of CKD. Therefore, this vulnerable patient population requires urgent attention and targeted interventions.

As CKD progresses, patients require renal replacement therapy for which dialysis is a treatment option. According to the GBD Chronic Kidney Disease Collaboration, there were approximately 3.14 million patients undergoing dialysis worldwide in 2017 [4]. By December 2023, data from the Chinese National Renal Data System (CNRDS) indicated that the total number of registered patients undergoing HD and peritoneal dialysis in mainland China had surpassed 1.06 million, including 916,647 patients undergoing HD [5]. Given the steady annual increase in patients undergoing HD, research on the adverse effects and complications associated with HD has garnered growing attention.

HD is widely used in clinical practice in China as a modality for renal replacement therapy. However, this is associated with numerous complications. Among these, PDF stands out as one of the most prevalent, distressing, and debilitating symptoms [6]. Unlike chronic and intradialytic fatigue, PDF is a unique and incapacitating sensation that occurs after dialysis sessions. Patients often describe it as feeling ‘worn out’ or ‘completely drained’ [7]. Notably, Standardized Outcomes in Nephrodialysis (SONG-HD) has identified PDF as a critical core outcome that must be routinely assessed in patients undergoing HD [8]. The prevalence of PDF varies widely, with estimates ranging from 20% to 86% [9–11]. A meta-analysis involving 12 studies and 1,215 patients undergoing HD reported an overall PDF prevalence of 61.0% [12]. This high prevalence underscores the significant clinical burden and adverse effects of PDF.

PDF exerts multidimensional adverse effects on patients undergoing HD, significantly affecting their physical, psychological, and social well-being. First, PDF in patients undergoing HD was independently associated with an increased risk of all-cause mortality, cardiac mortality, and the composite endpoint of the first cardiac hospitalization [13]. Second, patients undergoing HD are more likely to exhibit reduced engagement in daily activities, a diminished capacity to maintain interpersonal relationships, and difficulty meeting societal or familial expectations [7]. Finally, research has highlighted a particularly high prevalence of depression, anxiety, and cognitive impairment among patients undergoing HD with PDF [14]. Given these profound implications, the early identification of patients at an elevated risk of PDF is essential to mitigate adverse clinical outcomes and reduce mortality.

Numerous studies have attempted to develop predictive models of fatigue in patients undergoing HD. For instance, Qiao et al. used the Revised Piper Fatigue Scale to construct and internally validate a single-center prediction model for patients undergoing HD in China [15]. Similarly, Zhou et al. employed a Fatigue Scale to develop and internally validate a severe fatigue model based on a cohort of 243 patients undergoing HD in China [16]. However, these studies had notable limitations. First, they failed to differentiate between PDF and the broader cumulative experience of fatigue in their outcome indicators. Second, the sample sizes used in these models were relatively small, which raises concerns about their representativeness. Finally, traditional regression models are inadequate for capturing complex phenomena such as PDF. A possible solution for improving the prediction of PDF is the application of machine learning (ML) models.

ML models can account for the complex relationships among numerous factors and are not constrained by nonlinearity, thus enabling more accurate predictions [17]. Moreover, ML models can assess the predictive power of multiple factors and identify the most significant variables contributing to the outcomes. Meanwhile, ML is a rapidly growing field with expanding applications in various domains, including healthcare. To date, ML models, often referred to as ‘black boxes,’ have been rarely used in data analysis due to the difficulty in interpreting their outcomes [18,19]. Explainable artificial intelligence (XAI) focuses on making predictive models more understandable [20]. Naturally, this area is booming and continuously evolving, with recent advances providing a unified approach to explain the predictions made by any ML model in a post-hoc manner. One such tool is the Shapley additive explanation (SHAP), which incorporates concepts from game theory and offers both global and local explanations by integrating multiple methods [21].

To the best of our knowledge, no research has been conducted on the development of interpretable machine learning prediction models for PDF symptoms in patients undergoing HD. Additionally, few studies have focused on multi-dimensional predictors such as demographic characteristics, HD-related factors, laboratory parameters, and psychosocial factors. Through the selected multi-dimensional predictors and the use of explainable artificial intelligence (XAI) techniques, we further hypothesized that the interpretability of our models would allow healthcare providers to not only trust but also understand the decision-making process behind the model’s predictions, thereby facilitating their adoption in clinical practice. This could lead to better risk stratification and personalized management of patients undergoing HD, ultimately improving patient outcomes.

## 2. Methods

### 2.1. Study design and participants

This multicenter, cross-sectional, descriptive study was conducted in Taiyuan, the capital of Shanxi Province in northern China. We employed a two-stage sampling strategy. In the first stage, three districts were randomly selected from the six administrative districts in Taiyuan. In the second stage, patients on HD were recruited through convenience sampling from designated blood purification centers in hospitals within the selected districts. The study ultimately included patients from six blood purification centers, with 70% serving as the training set for model development and the remaining patients designated as the validation set.

A total of 1,281 patients undergoing HD who met the predefined inclusion and exclusion criteria were enrolled between May 2024 and November 2024. The inclusion criteria were as follows: (1) age ≥18 years; (2) undergoing regular HD treatment for at least 3 months with a frequency of three sessions per week; (3) each dialysis session lasts at least 3 h; and (4) providing informed consent voluntarily. The exclusion criteria were as follows: (1) presence of severe cardiovascular diseases, severe infections, malignancies, or other major organ disorders, and (2) pregnancy.

### 2.2. Features

Based on a comprehensive literature review and meta-analysis of the risk factors for PDF, in conjunction with recommendations from clinical HD experts, we identified 25 potential risk factors. These factors were categorized into four domains: (1) Demographic characteristics: age, body mass index (BMI), educational level, sex, and marital status; (2) HD-related factors: history of fistula surgery (HFS; defined as any surgical intervention beyond the initial arteriovenous fistula creation, including secondary fistula establishment, fistula repair, stenosis management, and angioplasty), comorbid conditions, constipation, pre-dialysis blood pressure, dietary intake during dialysis, and interdialytic hypotension (IDH); (3) Laboratory parameters: serum creatinine, albumin, cholesterol, calcium (Ca), phosphorus (P), potassium (K), hemoglobin, C-reactive protein (CRP), and ferritin levels; (4) Psychosocial factors: sleep quality, social support, psychological resilience, and appetite status. Data for all predictive variables were collected before the end of dialysis, and only the PDF was measured after dialysis.

The Pittsburgh Sleep Quality Index (PSQI), developed by Buysse et al. is a validated instrument designed to assess sleep quality over the preceding month [22]. This 19-item self-report questionnaire measures seven distinct sleep-related components: subjective sleep quality, sleep latency, sleep duration, habitual sleep efficiency, sleep disturbances, use of sleep medications, and daytime dysfunction. Each component is scored on a scale of 0–3, with higher scores indicating poorer sleep quality. The global PSQI score, ranging from 0 to 21, was calculated by summing all the component scores, with higher total scores corresponding to worse sleep quality. A total score > 5 points indicated poor sleep quality and potential sleep problems.

The Connor-Davidson Resilience Scale (CD-RISC-10), adapted by Campbell-Sills et al. is a unidimensional instrument designed to measure psychological resilience [23]. This scale consists of 10 items rated on a 5-point Likert scale ranging from 0 (‘never’) to 4 (‘always’), yielding a total score between 0 and 40. Higher total scores indicate greater resilience levels. In the present study, a cutoff score of 26 was established to differentiate resilience levels, with scores below 26 indicating low psychological resilience, and scores of 26 or above representing high psychological resilience.

The Simplified Nutritional Appetite Questionnaire (SNAQ), developed by the Council for Nutritional Strategies in Long-Term Care, is a validated screening tool designed to evaluate appetite and predict nutritional risk in patients with chronic illnesses [24]. As an abbreviated version of the Council on Nutrition Appetite Questionnaire (CNAQ), the SNAQ allows for the rapid and reliable identification of patients experiencing appetite reduction, which may predispose them to malnutrition and its associated complications. This instrument consists of four core items that assess (1) appetite changes over a specified period, (2) general interest in food, (3) specific food preferences or aversions, and (4) alterations in eating patterns. Each item is scored on a 5-point Likert scale, with a total score of ≤14 indicating a clinically significant risk of at least 5% body weight loss within 6 months.

The Social Support Rate Scale (SSRS), developed by Xiao et al. is designed to assess the level of social support that an individual receives [25]. The SSRS consists of 10 items that evaluate various dimensions of social support, including emotional, instrumental, and information support. The total possible score is 66, with higher scores indicating greater social support. Scores of 0–22 indicates low social support, 23–44 indicates moderate social support, and 45–66 indicates high social support.

### 2.3. Outcomes

The primary outcome was PDF. PDF assessment was conducted following the recommendations of Sklar et al. [26]. Patients were classified as having PDF if they spontaneously reported feeling fatigued in response to the open-ended question ‘Do you feel fatigued after dialysis?’ If the response was affirmative, each patient rated the intensity, duration, and frequency of fatigue on a scale of 1 to 5. Intensity was defined as the magnitude of the fatigue, duration as the length of time the fatigue persisted, and frequency as the number of times the fatigue occurred. PDF was considered present when the total score of the three indicators was ≥4. By using ROC-derived optimal cutoffs, 4.5 point was determined as the optimal cutoff value, and the sensitivity and specificity were 0.912 and 0.784 at this time. Considering the actual clinical application, rounding down to 4 points can effectively screen PDF, which confirms the robustness of the measurement tool.

### 2.4. Data processing

The K-nearest neighbor (KNN) algorithm with *K* = 5 in machine learning was applied to replace missing items for each scale. The missing data were missing Completely at random, and proportion of all variables was less than 20% because samples with more than 20% missing data were eliminated. The basic concept of KNN imputation is to predict missing values by utilizing neighboring samples with known values from the dataset [27]. Individuals reporting PDF represent the majority class, often resulting in unbalanced datasets. The imbalanced data posed a challenge in predicting PDF in this study because there were few examples of non-PDF cases from which to learn (1.71:1). Moreover, evaluation of the classification model showed a bias toward majority classes, which could lead to suboptimal performance. One technique for addressing imbalanced data is the Synthetic Minority Oversampling Technique (SMOTE), an algorithm that generates virtual replicates from the existing minority class in the training data, allowing prediction algorithms to learn from more examples [28]. The data were then divided into a training set (70% of the sample) and a test set (30% of the sample). The test set was used to evaluate the generalization ability of the models for unseen samples.

### 2.5. Feature selection

The factor selection process begins with Nested cross-validation with LASSO which employs a dual-loop framework to prevent overfitting during feature selection. The outer loop partitions data for performance evaluation, while the inner loop uses cross-validation to optimize LASSO’s regularization strength (λ). LASSO’s L1 penalty automatically eliminates redundant predictors by shrinking their coefficients to zero, yielding a sparse feature subset. Feature stability is assessed by frequency across outer folds.

### 2.6. Model development and performance metrics

**Figure 1.**
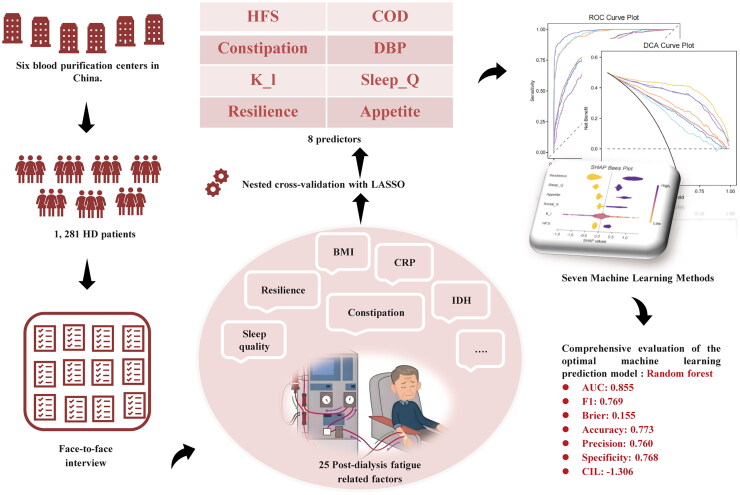
Study flow diagram.

Seven different machine learning models were employed in this study, as shown in Figure 1. The classifiers included a linear statistical method (logistic regression), two tree-based methods (decision tree and random forests), and four boosting methods (LightGBM, CatBoost, XGBoost, Gradient Boosting Tree). Machine learning models have been used extensively in various medical applications. To determine the optimal model hyperparameters, 5-fold cross-validation and grid search were applied to the training data. Model performance was evaluated using the following metrics: accuracy, recall, F1 score, AUC (Area Under the Curve), precision, and specificity. These metrics provide a comprehensive assessment of the ability of the model to classify both majority and minority classes, ensuring a balanced evaluation of its overall effectiveness, sensitivity, and ability to avoid false positives. In addition, SHAP methods based on cooperative game theory have been used to increase the interpretability of machine learning models. The SHAP value represents the deviation from the average predicted value for each case, predicted for each feature. This is a more granular method that can yield feature attributes for every individual instance, which can be beneficial for a more detailed and accurate understanding of the models.

### 2.7. Statistical analysis

In this study, features were summarized as means and standard deviations (SDs) for continuous variables, and counts and percentages for categorical variables. Differences between groups were assessed using independent t-tests (for continuous variables) and χ^2^ tests (for categorical variables). For the statistical analysis, we used R Project (version 4.0.5) and RStudio (version 4.0.2) and considered 95% confidence intervals (95% CI).

## 3. Results

### 3.1. Descriptive statistics

**Figure 2.**
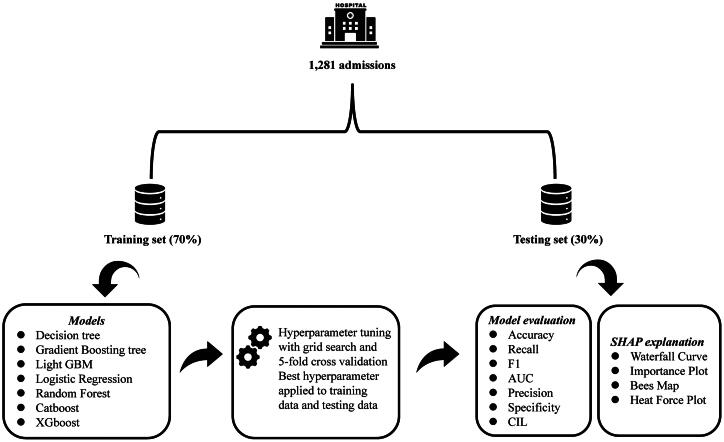
Heat map of the correlations between all the variables. The illustration reveals that there are no strong correlations among the variables (correlation coefficient < 0.7). PDF: postdialysis fatigue; DBP: diastolic blood pressure; SBP: systolic blood pressure; HFS: history of fistula surgery; BMI: body mass index; CRP: C-reactive protein; K_l: potassium; Ca_l: calcium; P_l: phosphorus; COD: combined other diseases; Eat: eating during dialysis; IDH: interdialytic hypotension; social_s: social support.

Descriptive statistics and general characteristics of the study variables are presented in Table 1. The final analysis included 1,281 patients undergoing HD (mean [SD] age, 54.48 [13.09] years; 836 [65.26%] male), of whom 808 (63.07%) developed PDF. There were multiple significant differences in scale scores between participants with and without PDF. Patients with PDF showed significantly lower pre-dialysis blood pressure, lower albumin levels, higher CRP levels, lower potassium ion levels, differences in educational level, more history of fistula surgery, more comorbidities, more constipation, more prone to hypotension during dialysis, more eating during dialysis, poorer sleep, lower social support, lower psychological resilience, and worse nutritional appetite. Regarding factor collinearity evaluation, the heatmap indicated no significant strong correlations between the variables (Figure 2).

**Table 1.** Baseline characteristics of patients undergoing HD.

Variable	Overall (*N* = 1281)	Non-PDF (*N* = 473)	PDF (*N* = 808)	*P*-value
Age (years)	54.48 ± 13.09	53.57 ± 13.21	55.02 ± 13.00	0.056
BMI (kg/m²)	23.14 ± 4.12	23.41 ± 4.21	22.98 ± 4.06	0.073
SBP (mmHg)	145.92 ± 17.46	147.69 ± 18.72	144.88 ± 16.61	0.007
DBP (mmHg)	78.41 ± 12.28	80.23 ± 12.77	77.34 ± 11.86	<0.001
Creatinine (μmol/L)	743.27 ± 255.94	751.98 ± 252.78	738.17 ± 257.80	0.349
Albumin (g/L)	38.59 ± 5.94	39.08 ± 3.50	38.30 ± 6.96	0.007
Cholesterol (mmol/L)	3.74 ± 0.95	3.79 ± 0.89	3.70 ± 0.98	0.107
Hemoglobin (g/L)	113.52 ± 16.33	114.37 ± 16.02	113.02 ± 16.50	0.148
CRP (mg/L)	11.49 ± 17.24	9.95 ± 13.52	12.39 ± 19.04	0.008
Ferritin (μmol/L)	158.29 ± 142.41	151.04 ± 154.58	162.54 ± 134.68	0.179
K_l (mmol/L)	4.78 ± 0.77	4.88 ± 0.79	4.72 ± 0.75	<0.001
P_l (mmol/L)	1.68 ± 0.51	1.69 ± 0.47	1.67 ± 0.53	0.487
Ca_l (mmol/L)	2.22 ± 0.21	2.22 ± 0.19	2.21 ± 0.21	0.571
Culture (Colleg and above)	207.00 (16.16%)	102.00 (21.56%)	105.00 (13.00%)	<0.001
Gender (male)	836.00 (65.26%)	324.00 (68.50%)	512.00 (63.37%)	0.063
Marital (married)	1,158.00 (90.40%)	418.00 (88.37%)	740.00 (91.58%)	0.060
HFS (true)	512.00 (39.97%)	166.00 (35.10%)	346.00 (42.82%)	0.006
COD (true)	793.00 (61.90%)	269.00 (56.87%)	524.00 (64.85%)	0.005
Constipation (true)	457.00 (35.68%)	124.00 (26.22%)	333.00 (41.21%)	<0.001
Eat (true)	910.00 (71.04%)	319.00 (67.44%)	591.00 (73.14%)	0.030
IDH (true)	279.00 (21.78%)	88.00 (18.60%)	191.00 (23.64%)	0.035
Sleep_Q (true)	750.00 (58.55%)	197.00 (41.65%)	553.00 (68.44%)	<0.001
Social_S (true)	340.00 (26.54%)	95.00 (20.08%)	245.00 (30.32%)	<0.001
Resilience (true)	651.00 (50.82%)	112.00 (23.68%)	539.00 (66.71%)	<0.001
Appetite (true)	422.00 (32.94%)	73.00 (15.43%)	349.00 (43.19%)	<0.001

BMI: body mass index; DBP: diastolic blood pressure; SBP: systolic blood pressure; HFS: history of fistula surgery; CRP: C-reactive protein; K_l: potassium; Ca_l: calcium; P_l: phosphorus; COD: combined other diseases; Eat: eating during dialysis; IDH: interdialytic hypotension; Sleep_Q: sleep quality; Social_S: social support.

### 3.2. Feature selection

To identify significant predictors of the target variable, we applied nested cross-validation combined with LASSO regression. The nested cross-validation process, involving 10-fold cross-validation, ensured model evaluation and selection were independent, avoiding data leakage and providing a robust assessment of model generalization. The cross-validation scores for each fold were as follows: −0.165, −0.178, −0.163, −0.165, −0.178, −0.186, −0.196, −0.164, −0.201, −0.155. The average score was −0.1758. The optimal regularization parameter, λ, determined through nested cross-validation was 0.019. This value balances model complexity and fitting accuracy, preventing overfitting while maintaining prediction control. The selected features with significant non-zero coefficients were: HFS, COD, Constipation, DBP, K_l, Sleep_Q, Resilience, and Appetite.

### 3.3. Model performance

**Figure 3.**
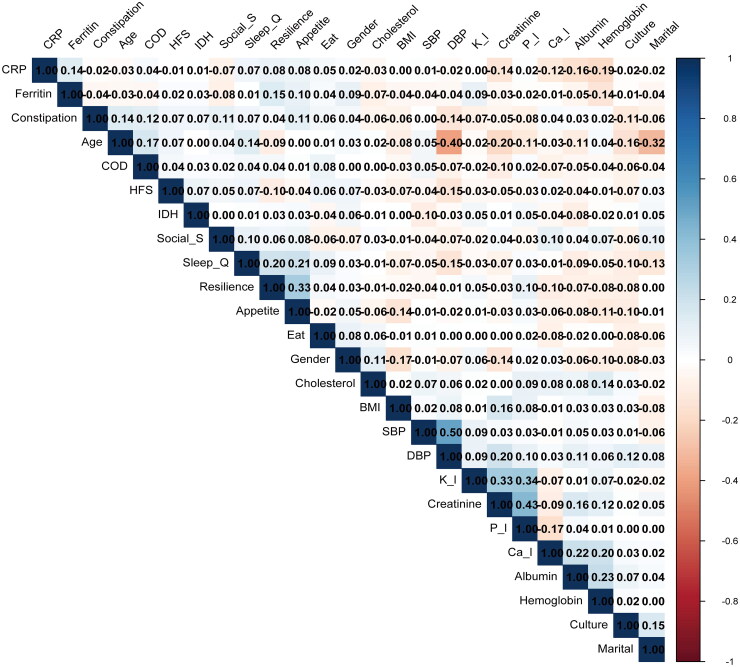
Training and test of PDF prediction model. (A) Receiver operating characteristic (ROC) curves for seven ML models in the training set. (B) Calibration curve of the seven ML models in the training set. (C) DCA curve for seven ML models in the training set. (D) Receiver operating characteristic (ROC) curves for seven ML models in the test set. (E) Calibration curve of the seven ML models in the test set. (F) DCA curve for seven ML models in the test set.

Six predictors were included, and seven different ML algorithms were used to construct PDF risk prediction models for patients undergoing HD. To ensure the stability and generalization ability of the model, a grid search combined with 5-fold cross validation was used to determine the optimal hyperparameters (Table 2). The performance of the model was verified based on the test set data, and the distinguishing performances of the seven models are listed in Table 3. The AUC of the LGBM and RF models for the test set were 0.993 and 0.983, respectively. Compared with that of other models, they exhibited higher discriminatory performance and clinical decision-making benefits, as shown in Figure 3(A–C). The accuracy, sensitivity, specificity, recall rate, and F1 score of these two models were strong. The accuracies and clinical applicabilities of the two models were evaluated using an external test set. The results showed that the RF model had better Brier score, with predicted results more consistent with the actual outcomes, and demonstrating greater net clinical benefits (Figure 3(D–F)). Finally, the results of DeLong ‘s test of the RF and LGBM models showed Z = −4.5127 and *p* < 0.001, which proved that there was a significant difference in AUC between the two models. Therefore, the RF model was selected as the final model for predicting the PDF risk in patients undergoing HD.

**Table 2.** Hyperparameter evaluation range for the ML models.

ML model	Hyperparameter	Search space	Selected value
Decision tree	max_depth	5, 10, 15, None	5
	min_samples_split	2, 5, 10	10
	min_samples_leaf	1, 2, 4	1
Gradient Boosting tree	n_estimators	50, 100, 150	150
	learning_rate	0.01, 0.1, 0.5	0.1
	max_depth	3, 5, 7	3
Light GBM	n_estimators	50, 100, 200	200
	learning_rate	0.01, 0.1, 0.5	0.01
	num_leaves	31, 63, 127	31
Logistic regression	C	0.01, 0.1, 1, 10	0.1
	solver	liblinear, saga	liblinear
Random Forest	n_estimators	50, 100, 200	200
	max_depth	10, 20, None	10
	min_samples_split	2, 5	2
	min_samples_leaf	1, 2	2
Catboost	iterations	50, 100, 200	200
	learning_rate	0.01, 0.1, 0.3	0.1
	depth	3, 5, 7	3
XGboost	n_estimators	50, 100, 200	50
	learning_rate	0.01, 0.1, 0.3	0.1
	max_depth	3, 5, 7	3

**Table 3.** Performance of ML models for predicting PDF.

Model Name		Accuracy	Recall	F1	AUC	Precision	Specificity	Brier	CIL
DesicionTree	Train	0.797	0.820	0.804	0.868	0.788	0.774	0.144	−1.507
	Test	0.775	0.804	0.776	0.828	0.750	0.748	0.165	−1.402
GBDT	Train	0.858	0.865	0.861	0.824	0.856	0.851	0.110	−1.911
	Test	0.779	0.774	0.772	0.859	0.771	0.784	0.152	−1.307
LGBM	Train	0.959	0.949	0.959	0.993	0.969	0.959	0.031	−3.022
	Test	0.779	0.770	0.771	0.838	0.773	0.788	0.163	−1.294
Logistic	Train	0.762	0.757	0.763	0.841	0.769	0.767	0.162	−1.187
	Test	0.761	0.744	0.751	0.845	0.757	0.776	0.159	−1.173
RF	Train	0.924	0.916	0.924	0.983	0.933	0.932	0.052	−2.421
	Test	0.773	0.779	0.769	0.855	0.760	0.768	0.155	−1.306
CatBoost	Train	0.798	0.813	0.803	0.877	0.793	0.783	0.143	−1.497
	Test	0.763	0.778	0.777	0.861	0.775	0.788	0.150	−1.332
XGB	Train	0.765	0.753	0.765	0.840	0.777	0.777	0.163	−1.199
	Test	0.754	0.719	0.739	0.846	0.761	0.788	0.159	−1.093

GBDT: gradient boosting decision tree; LGBM: light gradient boosting machine; Logistic: logistic regression; RF: random forest; CatBoost: categorical boosting; XGB: XGBoost; CIL: calibration-in-large.

### 3.4. Variable importance

**Figure 4.**
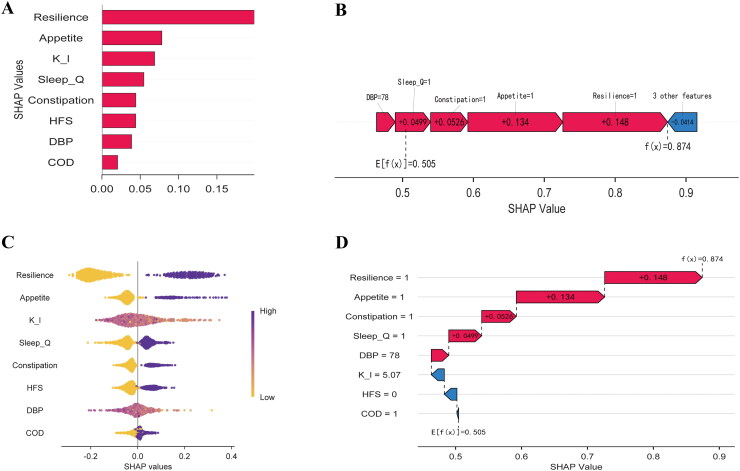
SHAP features for RF model. (A) SHAP feature importance. (B) SHAP force plot for explaining of individual’s prediction results. (C) Beeswarm summary plot. (D) SHAP waterfall plot for explaining of individual’s prediction results. K_l: potassium; Sleep_Q: sleep quality; HFS: history of fistula surgery; DBP: diastolic blood pressure; COD: combined other diseases.

Figure 4(A) shows the SHAP feature importance, calculated as the average absolute SHAP value per feature, for the RF model trained previously to predict the PDF. Features with large absolute shapley values are considered important. Resilience, appetite, potassium levels, sleep quality, constipation, HFS, DBP, and COD were the predictors that contributed the most to the model.

The SHAP values were calculated and plotted (Figure 4(C)). Resilience, appetite, potassium levels, sleep quality, constipation, HFS, DBP, and COD were the most important features distinguishing the PDF and non-PDF groups. Resilience showed positive SHAP values, indicating a positive correlation. This implies that patients diagnosed with poorer psychological resilience are more likely to develop PDF, as are those with poorer sleep quality, lower potassium levels, poorer nutritional appetite, and a history of fistula surgery, all of which contribute to a greater risk of developing PDF.

To explore the contribution of the predictors and clinical model applications, we randomly selected one patient from the dataset (Figure 4(B,D)). The results suggested that when the patient’s potassium level was 5.07 mmol/L, the HFS was positive, sleep was poor, nutritional appetite was poor, psychological resilience was low, and with constipation. The probability of PDF was 0.874 and model prediction was positive.

## 4. Discussion

The current study compared four ML models for predicting PDF among Chinese patients undergoing HD, incorporating a wide array of sociodemographic, HD-, laboratory-, and psychosocial-related variables. The importance of each predictor was examined using the best-performing ML model.

### 4.1. Prediction of PDF

A comparison of seven commonly used ML models revealed that the RF model was the most powerful in predicting PDF. The RF model built in this study demonstrated good performance, with a predictive accuracy of 0.924 and 0.773 in the training and test sets, respectively. The AUC of this model was 0.983 and 0.855 in the two sets. The performance of all models in the test set decreased, indicating that overfitting may have occurred. In subsequent studies, we will add more representative predictors to reduce the risk of overfitting. Even so, the RF model’s AUC score of 0.855 is an above-average score, suitable for most non-extreme tasks; however, there is room for improvement. Similarly, the RF model is also good in terms of Brier score and CIL performance (0.155 and −1.306). The RF prediction model demonstrates good calibration, with a calibration-in-the-large value of −1.306, suggesting that the model’s predicted probabilities are systematically lower than the true outcomes across the entire probability range, which is acceptable. The discrimination and calibration efficiency of the RF model are better in terms of comprehensive comparison. Given the seriousness of PDF, high sensitivity of the model is crucial. The sensitivity of the RF model in this study was 0.916 and 0.779 in both sets. The RF model is one of the most commonly used ML models for classification and regression [29]. It randomly combines results by constructing multiple decision trees. In general, the RF model demonstrates excellent accuracy for classification problems with high-dimensional features. Several previous studies have reported the use of ML models to predict complications in patients undergoing HD. For example, Dong et al. developed two machine learning-based models (IDH-A and IDH-B) using LightGBM to predict the intradialytic hypotension (IDH) risk in patients undergoing HD, demonstrating superior performance and interpretability compared to that of other models [30]. Hsieh et al. found that machine learning models, especially XGBoost, accurately predicted monthly iPTH levels in patients undergoing HD, with the best performance (AUROC 0.922) for predicting iPTH levels ≥600 pg/ml, potentially improving early identification of high-risk patients and reducing reliance on retrospective blood tests [31].

Existing clinical measures for identifying fatigue in patients undergoing HD are traditional regression models, which suffer from insufficient sample representativeness and fail to differentiate between PDF and the broader cumulative experience of fatigue in their outcome indicators. These models may also require clinical expertise to interpret results and present challenges for timely intervention. Thus, a computerized model that efficiently predicts the SI in large populations would improve the efficiency of PDF prevention. ML models can run automatically and the results may serve as complementary steps for enhancing the identification of individuals with PDF. Furthermore, Franklin et al. (2017) emphasized the need for a shift in the research focus from traditional modeling techniques to methods from the field of ML in future studies, as the predictive ability of traditional approaches has not improved in the past 50 years [32]. However, interpretable machine learning prediction models for PDF in patients undergoing HD are still lacking. The present study builds on the previous work by incorporating a large sample of Chinese patients undergoing HD. In addition, we developed ML models based on the observation and assessment of multidimensional predictors of patients undergoing HD. This ML model for PDF risk screening has the potential to be incorporated into regular health checkups conducted at blood purification centers.

### 4.2. Variable importance

The results of this study indicate that factors within multiple domains (i.e. individual, HD-related, laboratory parameters, and psychosocial factors) are important in predicting PDF in Chinese patients undergoing HD. Regarding psychosocial factors, the findings demonstrated that resilience was the most important contributor to PDF. Reportedly, higher psychological resilience can help alleviate fatigue symptoms in patients with chronic diseases [33]. This may be because individuals with higher resilience tend to have better emotional regulation and stress-coping strategies, which assist in managing negative emotions, such as anxiety and depression during dialysis, thereby reducing their impact on fatigue symptoms [34]. Additionally, resilience is associated with improved physiological recovery, as resilient individuals may be more effective at maintaining homeostasis, such as stable blood pressure, heart rate, and hormonal levels, which can help mitigate PDF [35]. Finally, higher resilience is linked to greater self-efficacy, leading patients to engage in healthier behaviors such as regular exercise, proper nutrition, and medication adherence, all of which can reduce the severity of fatigue symptoms. Therefore, to enhance the psychological resilience of patients on HD, clinicians should incorporate strategies such as cognitive-behavioral therapy (CBT) for emotional regulation, encourage the establishment of strong social support networks, promote regular physical activity to improve both physical and mental health, and introduce meditation and relaxation techniques to reduce stress and anxiety [36].

Electrolyte imbalances are common in patients undergoing dialysis and can be exacerbated by poor digestion, further increasing PDF. Our study found that hypokalemia in patients undergoing HD can exacerbate PDF by impairing muscle function, causing weakness and reduced energy levels, owing to the essential role of potassium in muscle contraction and cellular energy production [37]. Therefore, healthcare providers should proactively address constipation, monitor and care for fistula sites, and manage hypokalemia through regular assessments, dietary adjustments, and patient education to reduce PDF and improve patient well-being.

Maintaining a proper nutritional status in patients undergoing HD is crucial for their health and quality of life. Our results showed a statistically significant association between lower nutritional appetite and PDF, consistent with the findings of Salamon et al. [38]. The relationship between nutritional appetite and PDF can be explained by several mechanisms. Insufficient energy supply due to poor appetite or malnutrition leads to decreased physical strength and increased fatigue, and impaired immune function resulting from malnutrition makes patients more susceptible to infections and inflammation, further exacerbating fatigue [39]. Additionally, psychological issues, such as depression and anxiety, which are often associated with reduced appetite, can create a vicious cycle that worsens both appetite and fatigue [40]. As clinical nurses, collaborating with dietitians to create personalized dietary plans can help patients undergoing HD overcome appetite loss by offering energy-dense, protein-rich foods that comply with dietary restrictions and enhance their nutritional intake. At the same time, the relationship between constipation and PDF needs to be further explored.

Our results confirmed the relationship between low sleep quality and PDF. Zakariya et al. found that 56.9% of patients undergoing HD experience sleep disorders [41], which are associated with more severe fatigue; this finding is further supported by George et al. who identified a significant positive correlation between sleep disturbances and PDF [42]. Poor sleep quality may impair physiological recovery by increasing inflammation and disrupting the immune function, thereby exacerbating PDF. Additionally, inadequate sleep hinders energy restoration and affects the neuroendocrine system, elevating stress hormones such as cortisol, and disrupting neurotransmitter balance, further contributing to PDF [43]. Therefore, improving sleep quality in patients undergoing dialysis is crucial for enhancing physiological recovery, energy restoration, and immune function, which in turn reduces PDF. Clinicians should address sleep disturbances by optimizing the dialysis processes, providing relaxation techniques, and implementing targeted interventions to support both physical and mental well-being.

Interestingly, our results indicated that patients undergoing HD with constipation were more likely to develop PDF. However, some studies suggest that HD patients with PDF are more prone to constipation [44]. Previous reports also suggest that constipation can cause abdominal distension, leading to discomfort and increased intra-abdominal pressure [45]. This may exacerbate fatigue by affecting overall physical comfort and contributing to feelings of bloating and heaviness, which often worsen after dialysis sessions [46]. Additionally, chronic constipation can interfere with proper digestion and nutrient absorption, leading to deficiencies in essential nutrients, such as potassium and calcium, both of which can contribute to fatigue [37]. Therefore, research on constipation and PDF remains to be further explored.

In this study, we defined any surgery other than the initial establishment of a fistula as history of surgery. Our findings revealed an association between PDF symptoms and HD fistula surgery. Patients who undergo reoperation for an internal fistula may experience complications such as dysfunction, thrombosis, infection, stenosis, pseudoaneurysm, difficulties with puncture, and insufficient blood flow. Many of these issues can lead to inadequate dialysis adequacy in patients undergoing HD, potentially contributing to development of PDF. At the same time, our study found that patients with abnormal diastolic blood pressure before dialysis and other diseases were more likely to have PDF. This reminds medical workers in the blood purification center to pay extra attention to such patients and implement graded nursing and targeted nursing measures to prevent the occurrence of PDF.

### 4.3. Strengths, limitations, and future directions

Compared to that of previous research, this study has several major strengths. First, the predictors of PDF covered multiple domains, including sociodemographic, HD-, laboratory-, and psychosocial-related variables, thereby enabling the identification of robust and reliable predictors of PDF. Second, this study used multiple information sources from six hospitals in Taiyuan, China, which enhanced the credibility of the results. Third, this study developed ML models to predict PDF based on interpretable machine-learning algorithms. Fourth, our study successfully differentiated PDF from the broader cumulative experience of fatigue in terms of outcome indicators, including chronic and interdialytic fatigue.

Despite these strengths, this study has some limitations. First, the sample included patients undergoing HD from Northern China, which may have limited the generalizability of the findings. Therefore, replication is required for different samples. Second, although this study incorporated a wide array of sociodemographic, HD-, laboratory-, and psychosocial variables, other important factors may have been overlooked. Future research should endeavor to include additional information, such as genetic factors, functional magnetic resonance imaging (fMRI), and ultrasound. Third, the cross-sectional design may have introduced subjective and selection biases. Consequently, multicenter international prospective studies should be conducted in the future. Fourth, the constructed prediction model was not externally validated. Therefore, subsequent research should perform external validation to test the generalizability of the model. Consequently, the findings should be interpreted cautiously and professionals should exercise particular care when applying these results in clinical practice.

## 5. Conclusion

This study compared seven ML models to predict PDF in Chinese patients undergoing HD. The results indicated that the RF model outperformed the other six ML models in terms of accuracy and overall performance. Furthermore, this study identified resilience, appetite, potassium levels, sleep quality, constipation, HFS, DBP, and COD as having the highest values for predicting PDF. The effectiveness of the RF model underscores the potential of ML models as convenient screening and evaluation tools for PDF risk assessment. Such assessments offer opportunities for early intervention and the prevention of further risks.

## Supplementary Material

subfigures.zip

## Data Availability

The datasets generated and/or analyzed during the current study are not publicly available owing to ongoing analysis addressing other research questions, but are available from the corresponding author on reasonable request.
