# Supplementary figures and images for: Machine learning to predict postdialysis fatigue in patients undergoing hemodialysis
Source: Ren Fail. 2025 Jul 27;47(1):2529452. doi: 10.1080/0886022X.2025.2529452 (PMC12302430; doi:10.1080/0886022X.2025.2529452)

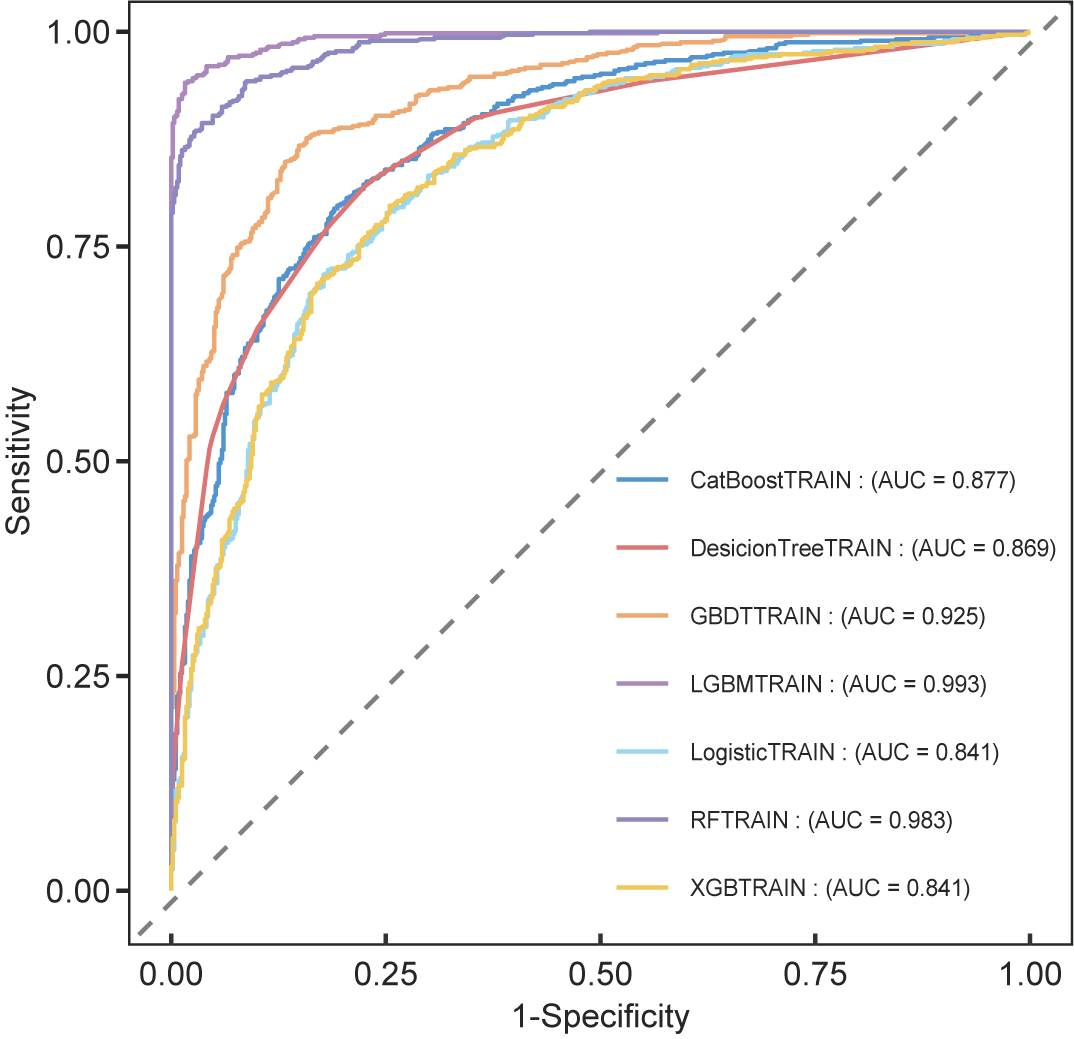

Supplement: subfigures.zip [file IRNF_A_2529452_SM1938.zip › subfigures/fig4a.tif]

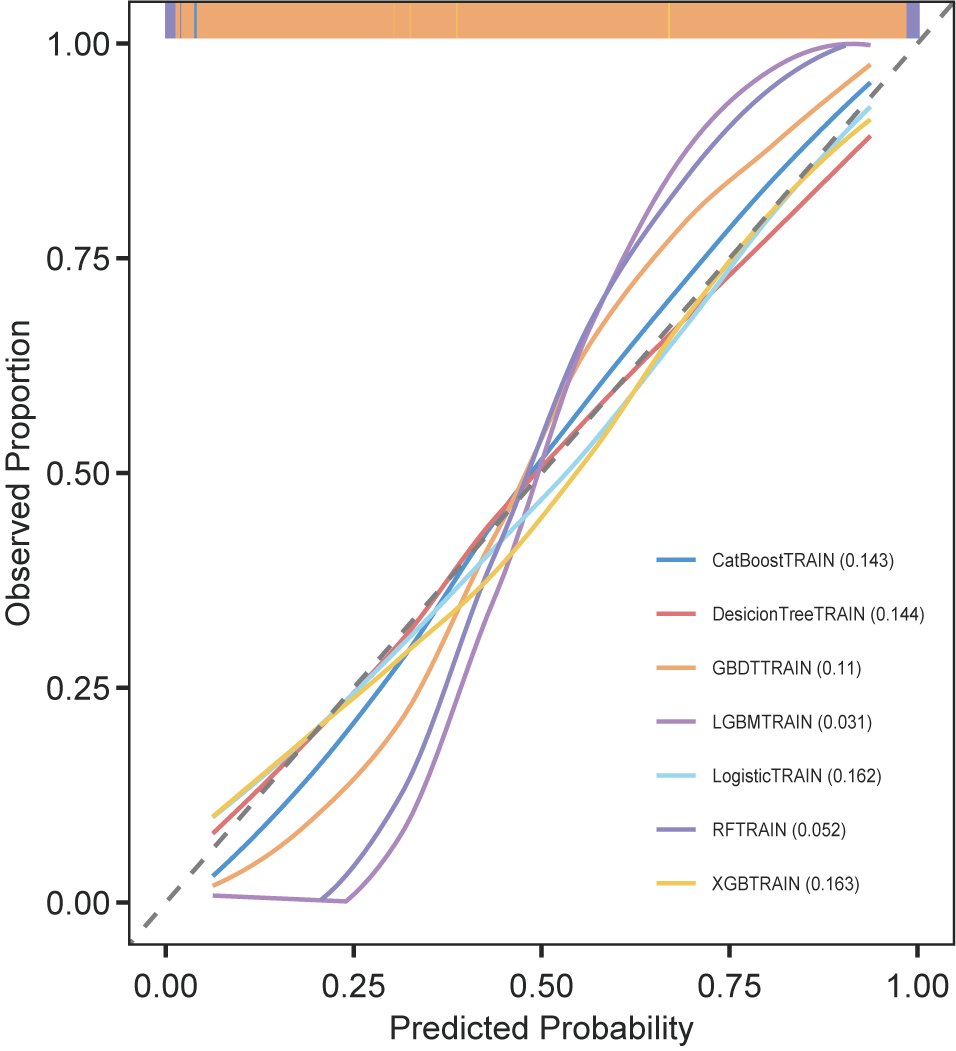

Supplement: subfigures.zip [file IRNF_A_2529452_SM1938.zip › subfigures/fig4b.tif]

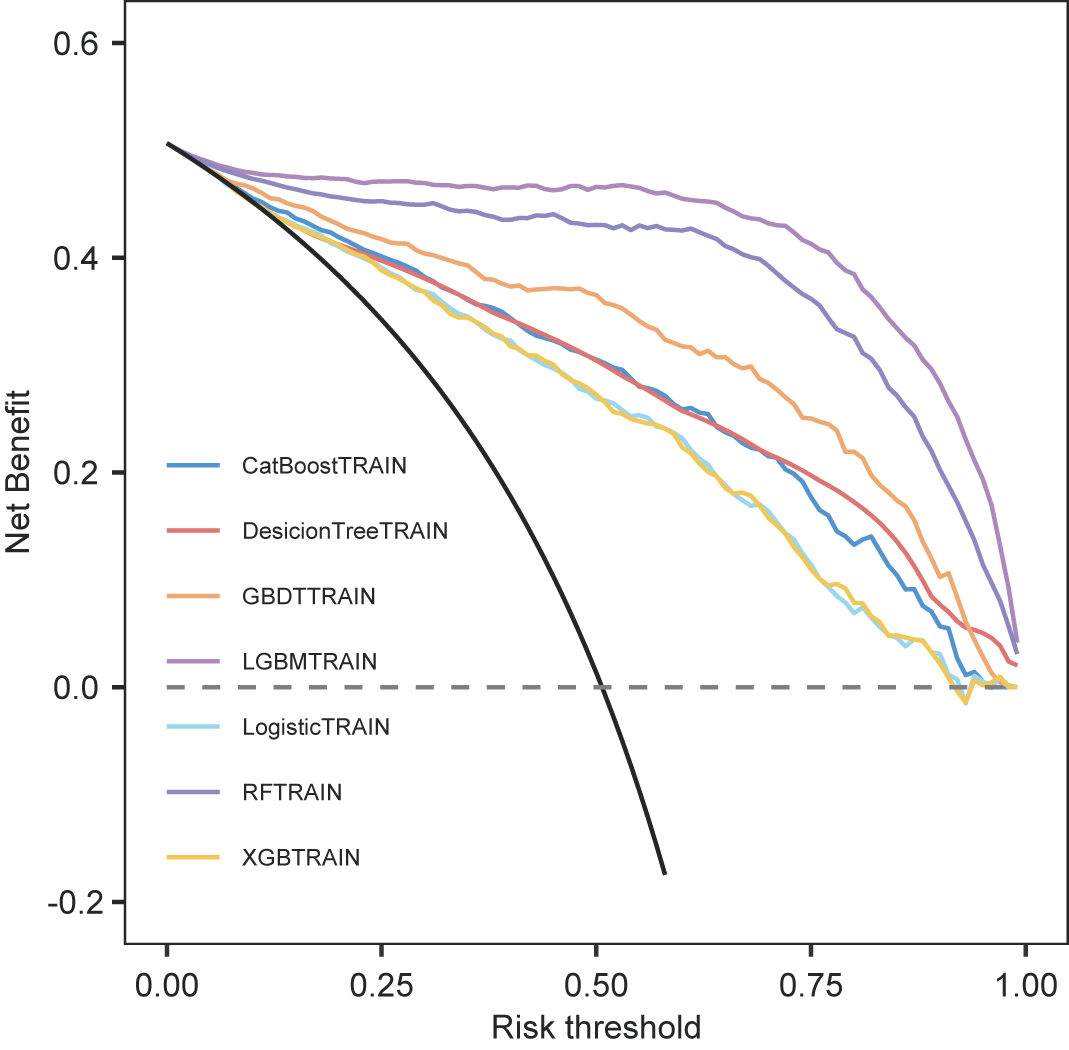

Supplement: subfigures.zip [file IRNF_A_2529452_SM1938.zip › subfigures/fig4c.tif]

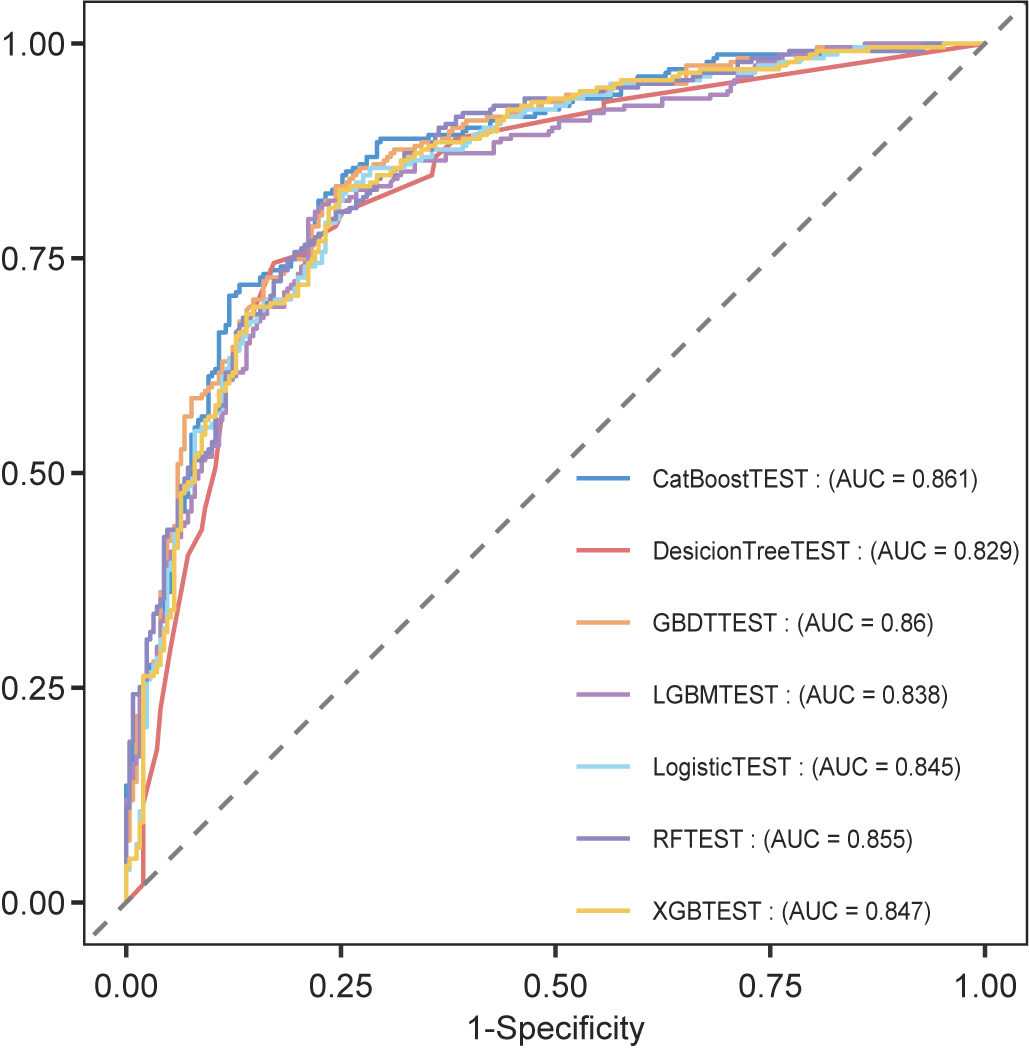

Supplement: subfigures.zip [file IRNF_A_2529452_SM1938.zip › subfigures/fig4d.tif]

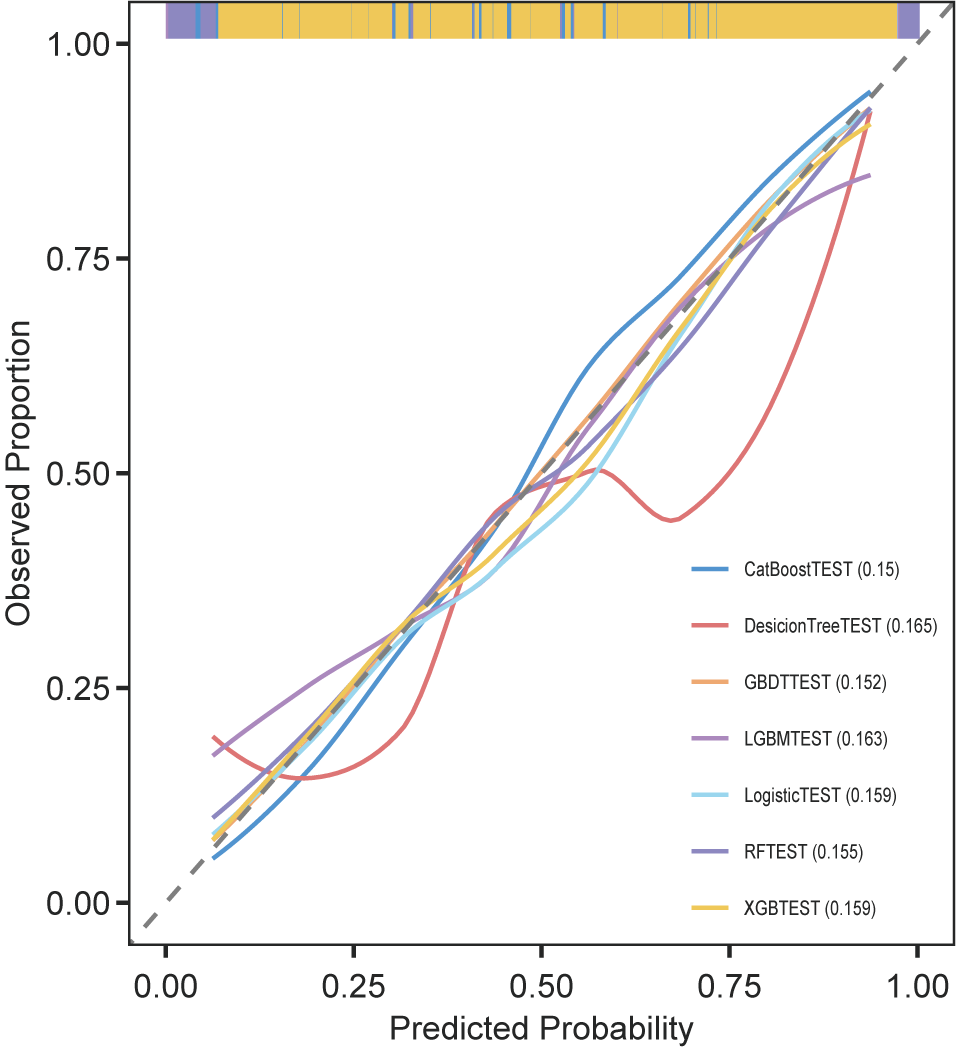

Supplement: subfigures.zip [file IRNF_A_2529452_SM1938.zip › subfigures/fig4e.tif]

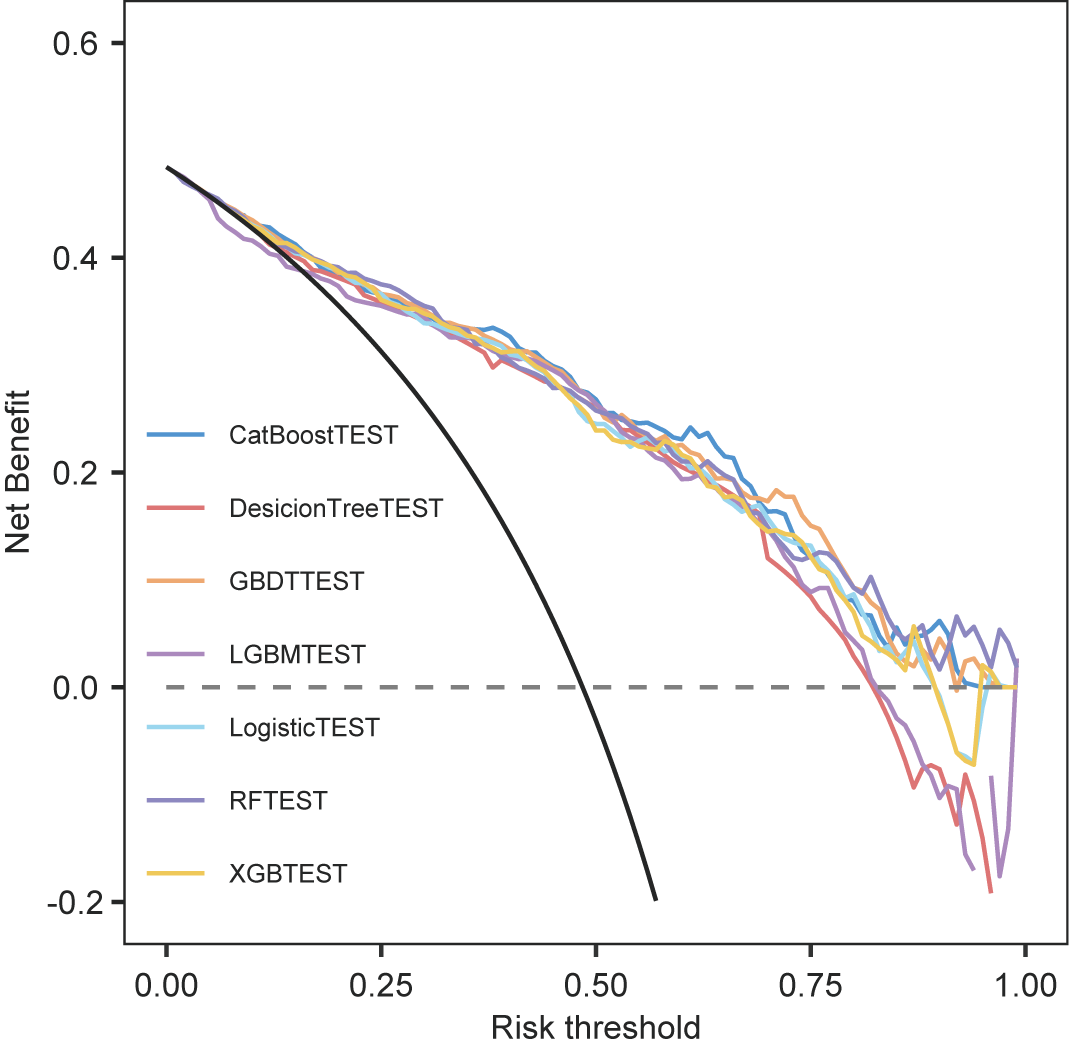

Supplement: subfigures.zip [file IRNF_A_2529452_SM1938.zip › subfigures/fig4f.tif]

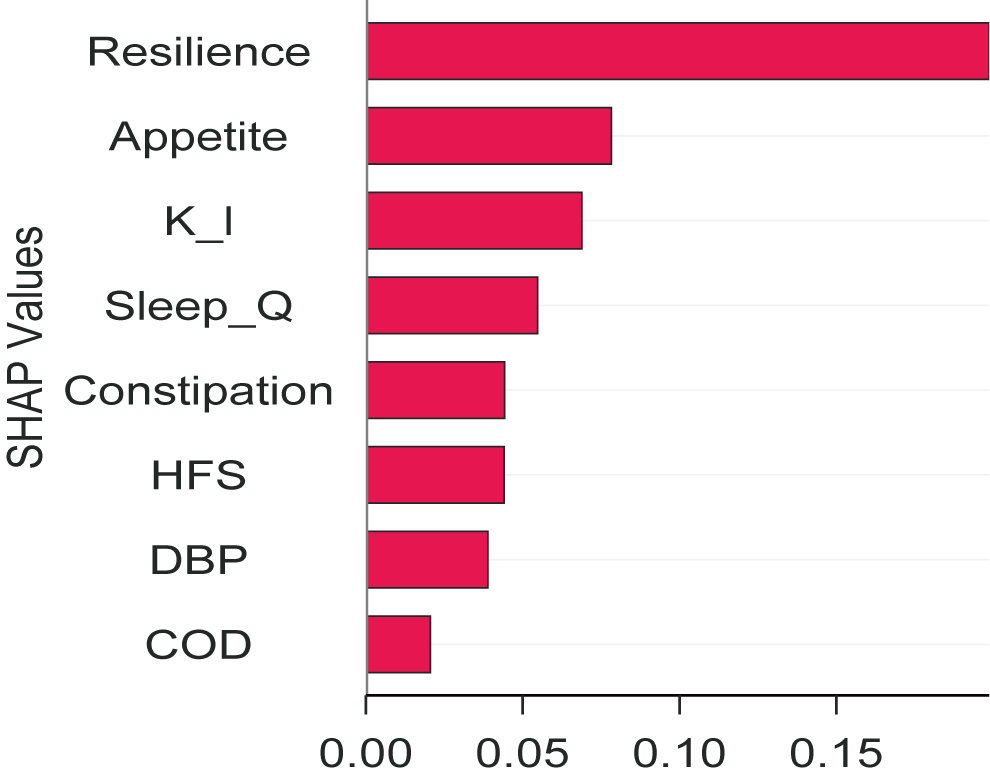

Supplement: subfigures.zip [file IRNF_A_2529452_SM1938.zip › subfigures/fig5a.tif]

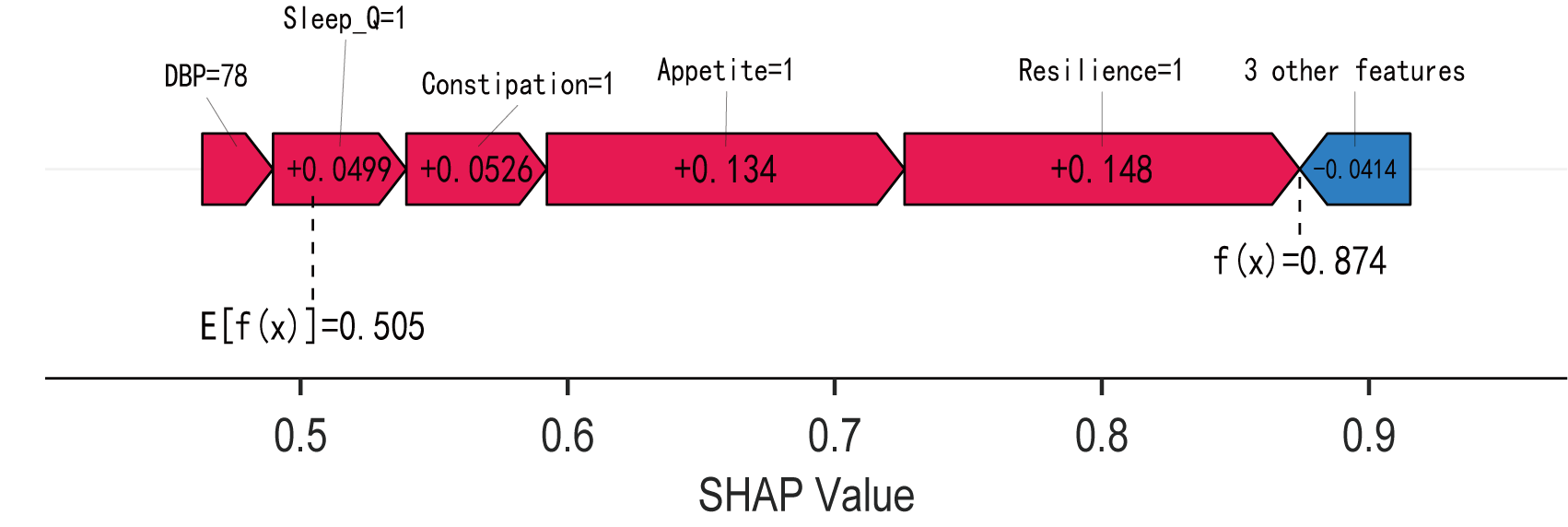

Supplement: subfigures.zip [file IRNF_A_2529452_SM1938.zip › subfigures/fig5b.tif]

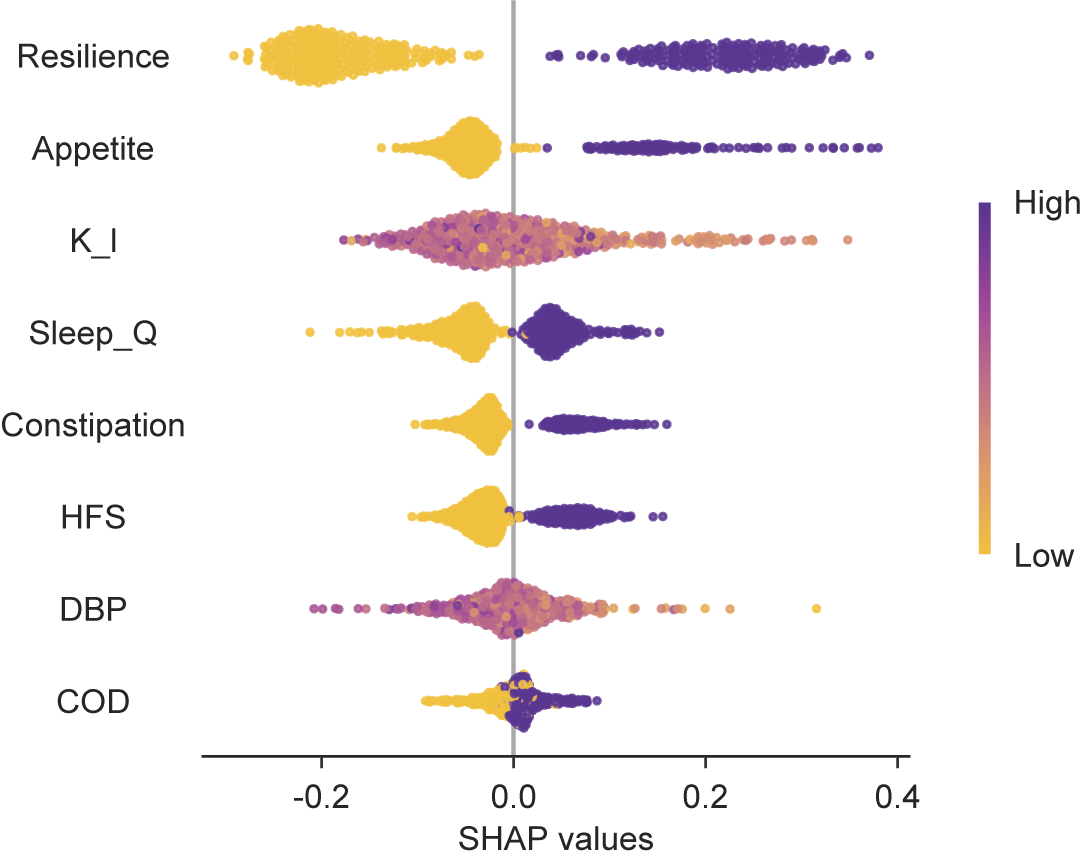

Supplement: subfigures.zip [file IRNF_A_2529452_SM1938.zip › subfigures/fig5c.tif]

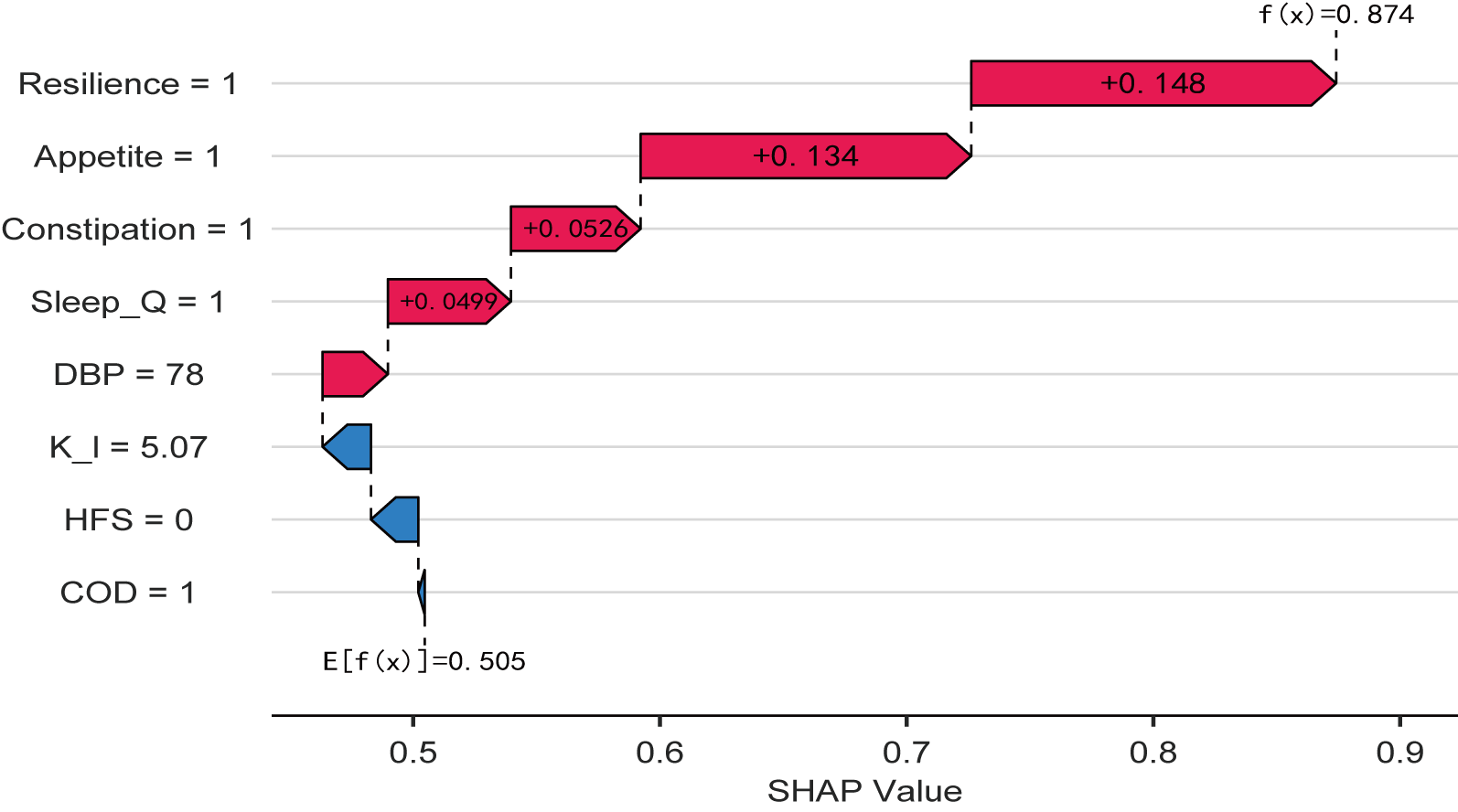

Supplement: subfigures.zip [file IRNF_A_2529452_SM1938.zip › subfigures/fig5d.tif]
